# Developing Gut Microbiota Exerts Colonisation Resistance to *Clostridium* (syn. *Clostridioides*) *difficile* in Piglets

**DOI:** 10.3390/microorganisms7080218

**Published:** 2019-07-26

**Authors:** Łukasz Grześkowiak, Temesgen Hailemariam Dadi, Jürgen Zentek, Wilfried Vahjen

**Affiliations:** Institute of Animal Nutrition, Freie Universität Berlin, Königin-Luise Strasse 49, 14195 Berlin, Germany

**Keywords:** *Clostridium difficile*, *Clostridioides*, gut microbiota, sequencing, qPCR, enrichment, pig, suckling, weaned, sow

## Abstract

*Clostridium* (syn. *Clostridioides*) *difficile* is considered a pioneer colonizer and may cause gut infection in neonatal piglets. The aim of this study was to explore the microbiota-*C. difficile* associations in pigs. We used the DNA from the faeces of four sows collected during the periparturient period and from two to three of their piglets (collected weekly until nine weeks of age) for the determination of bacterial community composition (sequencing) and *C. difficile* concentration (qPCR). Furthermore, *C. difficile*-negative faeces were enriched in a growth medium, followed by qPCR to verify the presence of this bacterium. *Clostridium*-sensu-stricto-1 and *Lactobacillus* spp. predominated the gut microbiota of the sows and their offspring. *C. difficile* was detected at least once in the faeces of all sows during the entire sampling period, albeit at low concentrations. Suckling piglets harboured *C. difficile* in high concentrations (up to log_10_ 9.29 copy number/g faeces), which gradually decreased as the piglets aged. Enrichment revealed the presence of *C. difficile* in previously *C. difficile*-negative sow and offspring faeces. In suckling piglets, the *C. difficile* level was negatively correlated with carbohydrate-fermenting bacteria, and it was positively associated with potential pathogens. Shannon and richness diversity indices were negatively associated with the *C. difficile* counts in suckling piglets. This study showed that gut microbiota seems to set conditions for colonisation resistance against *C. difficile* in the offspring. However, this conclusion requires further research to include host-specific factors.

## 1. Introduction

The gut microbiota of pigs develops rapidly after birth, where the early gut colonisers between one and three days of age originate from the mother and environment [1]. This developing and yet fragile ecosystem is exposed to numerous environmental factors, which may shape its composition and activity until it becomes relatively more stable. The association between mother and offspring gut microbiota during early life is a critical factor for the subsequent succession of intestinal commensal bacteria, immune development, and gut digestive functions [2,3]. The early neonatal phase also seems to determine the microbial profile and intestinal health later in the offspring’s life [4,5]. However, the aspect of mother–offspring association and its effect on early “microbial programming”, including colonisation resistance to pathogens in pigs has not been studied in detail.

*Clostridium* (syn. *Clostridioides*) *difficile* is a spore-forming microorganism that inhabits the human and animal gut, including pigs, which are also potential reservoirs of this bacterium in the environment [6,7]. The shedding of this bacterium by farm animals is of concern regarding the zoonotic transmission to farm workers and food products [8,9]. *C. difficile* is one of the pioneer colonisers in neonatal piglets. *C. difficile* and its toxins are occasionally detected in gestating and lactating sows [10], indicating that the sows could most likely be a primary source of *C. difficile* spores for their offspring, including virulent ribotypes [11,12]. However, this has yet to be verified. Indeed, virulent ribotypes of *C. difficile* and its toxins are commonly detected in the faeces of new-born piglets [9,10], where toxins may lead to spontaneous gut infection in neonatal piglets. However, it is unclear why and under what conditions piglets get sick or get asymptomatically colonised by virulent ribotypes. High levels of *C. difficile* and their toxins have been found in neonatal piglets without demonstrating symptoms of infection [10]. As in humans, the pathogenesis of *C. difficile* infection (CDI) is most likely multifactorial, and thus, accompanied by co-infections with other pathogens or a predisposing intestinal dysbiosis caused by antimicrobials [13,14]. Interestingly, *C. difficile* is rarely detected, and infection has not been reported in older pigs, including sows [10,12,15]. It seems that any disruption of the natural colonisation process or perturbances of the intestinal ecosystem could increase the chances of piglets developing CDI. This is well established in humans suffering from CDI, where antibiotic treatment is considered the main cause of recurrent CDI [13,14]. The phenomenon of colonisation resistance and susceptibility to CDI in piglets still needs to be elucidated.

The aim of this study was to explore the possible associations between gut microbiota and *C. difficile* colonisation in sows and their offspring.

## 2. Materials and Methods 

### 2.1. Animals and Sampling

The institutional and national guidelines for the care and use of animals were followed. The sampling of faecal material from sows, suckling (1–4 weeks old), and weaned piglets (5–9 weeks old) required no ethical approval. Furthermore, the weaned piglets belonged to a control group of another trial approved by the Landesamt für Gesundheit und Soziales, Berlin on the date 03 April 2018 (identification number G0042/18). This study was conducted in the experimental pig facilities at the Institute of Animal Nutrition at the Freie Universität Berlin in Berlin, Germany. The institute has a sow breeding facility and its piglets are regularly produced (German Landrace). Each sow and their piglets were individually housed in commercial flat-deck pens. Gestating sows were fed a standard gestating (per kg of dry matter: 11.9 MJ of metabolisable energy, 16.0% crude protein, 7.5% crude fibre) and a standard lactating (13.0 MJ of metabolisable energy, 17.9% crude protein, 5.0% crude fibre) diet after farrowing, according to standard recommendations by the GfE 2006 [16]. At weaning, the piglets were separated from their mothers and placed in pens (two pigs/pen) based on the plan from another trial. All animals remained healthy during the sampling period. 

Faecal samples from four sows were collected at two and one months, and one week before, and one week after birth. A total of 102 faecal samples from randomly chosen offspring (two to three piglets/sow) from the four sows were collected weekly until nine weeks of age. 

### 2.2. DNA Extraction

DNA from faecal samples (0.2 g) was extracted using the QIAamp DNA Stool Mini Kit (Qiagen, Hilden, Germany) following the manufacturer’s instructions. The extraction protocol was preceded by repeated bead-beating on a FastPrep-24™ 5G homogeniser (MP Biomedicals, LLC, Santa Ana, CA, USA) to increase DNA extraction performance from spore-forming [17] and possibly the gram-positive bacteria.

### 2.3. Sequencing and Computational Data Analysis

DNA extracts were subjected to amplicon sequencing using an Illumina NextSeq500 sequencer (LGC, Berlin, Germany). Using a universal primer set, the 341F-785R V3-V4 region of the 16S rDNA was targeted and sequenced, resulting in 4.5 million sequence-read-pairs. The forward and reverse reads had a median length of 197 and 264 nucleotides, respectively. The forward and reverse reads were also combined using the BBMerge tool (version 34.48) [18]. The median length of the combined reads was 404 nucleotides. After demultiplexing, the resulting 16S-rDNA sequences of 102 samples were analysed using the QIIME2 pipeline [19] to determine the microbial community profiles of each sample. Specifically, quality control was performed using the DADA2 [20] routine within QIMME2. In the quality control process, chimeric sequences were removed, and regions of sequences with low-quality scores were truncated. Then, the exact amplicon sequence variants [21] and their respective counts in each sample were determined using DADA2. Sequence variants with total counts that were less than five sequences were excluded from further analysis as they could be contaminants. Such sequences were relevant to a maximum of five samples. To account for the uneven distribution of sequences within samples, normalisation was done through rearification [22]. After rearification, all samples were represented equally by 10,000 sequences to accommodate the sample with the lest number of sequences. Taxonomic assignment of the exact amplicon sequence variants was done using QIIME2’s feature classifier [23,24] together with the SILVA SSU database [25] (release 132). Subsequent genus-level taxonomic profiles were generated based on the assignment of sequences and their corresponding counts. Further statistical analysis was done using RStudio [26] and KNIME [27]. Specifically, R packages: stats, ggtree, and Hmisc were used to perform principal component analysis (PCA), phylogenetic, and correlation analyses, respectively. Principal components were calculated via singular value decomposition of the data matrix as defined by *prcmp* method under the R package stats. Differences for the diversity indices were calculated using the Kruskal-Wallis H test (significance at *p* ≤ 0.05), and associations between the *C. difficile* counts and microbiota abundance (correlations with significance *p* < 0.1 and rho > 0.5 were considered) and between microbial diversity indices were assessed using the Pearson’s r correlation analysis procedure (*n* = 31). Given the minimal abundance of sequences belonging to *C. difficile*, no associations could be made between the qPCR results and the 16S rDNA sequencing-based results.

### 2.4. qPCR and Sample Enrichment

DNA extracts were also subjected to qPCR analyses. For qPCR, primer sequences for the specific detection of the *C. difficile* 16S rDNA included Cdiff-16S-1f (5′-TTGAGCGATTTACTTCGGTAAAGA-3′) and Cdiff-16S-1r (5′-CCATCCTGTACTGGCTCACCT-3′). The master mix consisted of Brilliant II SYBR Green QPCR Master Mix with Low ROX (Stratagene, San Diego, CA, USA). An AriaMx Real-Time PCR (Agilent Technologies Inc., Santa Clara, CA, USA) was used for PCR amplification and fluorescent data collection. Genomic DNA extracted from the *C. difficile* was used to construct a calibration curve and it served as a positive control. A qPCR reaction mixture where the DNA template was replaced with deionised water served as a negative control. The amplification programme and data collection were performed as previously described in Reference [17].

Faecal samples from animals that were negative for *C. difficile* as assessed by qPCR (no *C*_t_ or distinct melting curve) were enriched in a growth medium composed of a brain-heart-infusion broth, yeast extract, l-cysteine, 0.1% sodium taurocholate, 25% cycloserine, and 1.6% cefoxitin. Faecal suspensions (10% *w*/*v*) were inoculated into the medium, homogenised by mixing, and then incubated anaerobically at 37 °C for 48 h. After that, the DNA extraction from 0.2 g of each sample was followed by the qPCR procedure described above, to verify the presence of *C. difficile*.

## 3. Results

### 3.1. Bacterial Community Development

**Figure 1:**
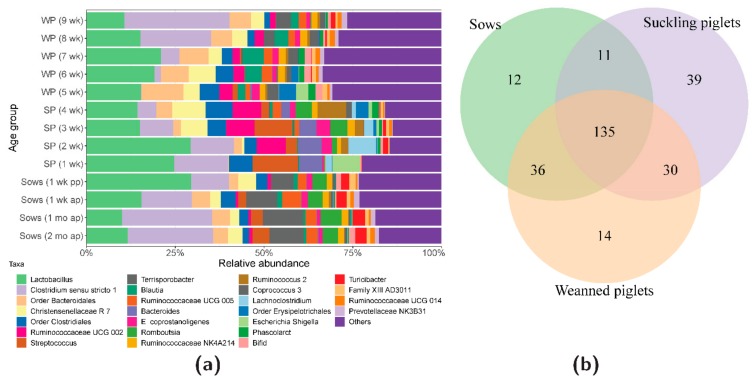
(**a**) Stacked bar plots showing the average percentage of bacterial taxa in sow and offspring faeces over time, (**b**) and a Venn diagram depicting the number of bacterial taxa that were unique and shared between sows, suckling, and weaned piglets, as analysed by the 16S rDNA sequencing.

In the individual animal samples, we found between 20 and 120 bacterial taxa. The total number of identified bacterial taxa was 277, of which the 25 dominant taxa were displayed in stacked bar plots. There were 252 non-specified bacterial taxa (Figure 1a).

Taxa of *Clostridium* sensu stricto 1 and *Lactobacillus* predominated the gut microbiota of the sows and their offspring throughout the sampling period. The sows gut microbiota was characterised by a high abundance of *Clostridium* sensu stricto 1, *Lactobacillus* spp., *Bacteroidales*, *Christensenellaceae* R7, *Clostridiales*, *Streptococcus* spp., *Terrisporobacter* spp., *Blautia* spp., *Ruminococcaceae* UCG005, *E. coprostanoligenes*, *Romboulsia* spp., and *Turicibacter* spp. In one-week-old piglets, *Lactobacillus* spp., *Clostridium* sensu stricto 1, *Clostridiales*, *Streptococcus* spp., *Bacteroides* spp., and *Escherichia-Shigella* taxa dominated the gut microbiota.

From the second week onwards, bacterial taxa belonging to *Bacteroidales*, *Ruminococcaceae* UCG 002, *Christensenellaceae* R7 and *Romboutsia* spp., *Ruminococcaceae* NK4A214, and *Ruminococcus* 2 spp. increased in abundance, whilst *Escherichia-Shigella* diminished. From the third week onwards, *Christensenellaceae* R7 increased in abundance, and from the fifth week onwards (beginning of weaning), taxa of *Ruminococcus* 2 spp. and *Lachnoclostridium* spp. diminished and *Prevotellaceae* NK3B31 appeared. In that week, bacterial taxa belonging to *Clostridium* sensu stricto 1 were not detected, whilst *Escherichia-Shigella* reappeared. Between the sixth and ninth weeks, the gut microbiota was dominated by *Lactobacillus* spp., *Clostridium* sensu stricto 1, *Bacteroidales*, *Christensenellaceae* R7, *Clostridiales*, *Ruminococcaceae ae* UCG002, *Terrisporobacter* spp., *Blautia* spp., *Ruminococcaceae* UCG005, *E. coprostanoligenes*, *Ruminococcaceae* NK4A214, and *Coprococcus* 3. The relative abundance of bacterial taxa in the sow and offspring faeces over time are shown in Figure 1a.

The sows, suckling, and weaned piglets shared 135 bacterial taxa, whilst the sows and suckling piglets shared 11 taxa, the suckling and weaned piglets shared 30 taxa, and the weaned piglets and sows shared 36 taxa (Figure 1b). Sows microbiota was unique for *Lachnospiraceae*, *Anaerococcus* spp., and *Lachnoclostridium* spp. Suckling piglets were exclusively colonised by *Actinomyces* spp. and *Actinobacillus* spp., while *Clostridium* sensu stricto 6 and *Anaerostipes* spp. were only found in the weaned piglets. Common bacteria in the sows and suckling piglets mainly belonged to *Peptostreptococcus* spp., *Erysipelatoclostridium* spp., *Luteococcus* spp., *Arcanobacterium* spp., *Corynebacterium* spp., *Succiniclasticum* spp., *Cerasicoccus* spp., *Pediococcus* spp., *Finegoldia* spp., *Kocuria* spp., and *Cutibacterium* spp. Both the suckling and weaned piglets were colonised by *Cloacibacillus* spp. and *Chlamydia* spp., whilst the weaned piglets and sows shared microbiota belonging to *Ruminococcaceae* UCG 008 and *Agathobacter* spp. amongst others. Supplementary Table S1 shows a list of all the bacterial taxa that were unique and shared between sows, suckling, and weaned piglets.

### 3.2. Bacterial Diversity Analyses

**Figure 2:**
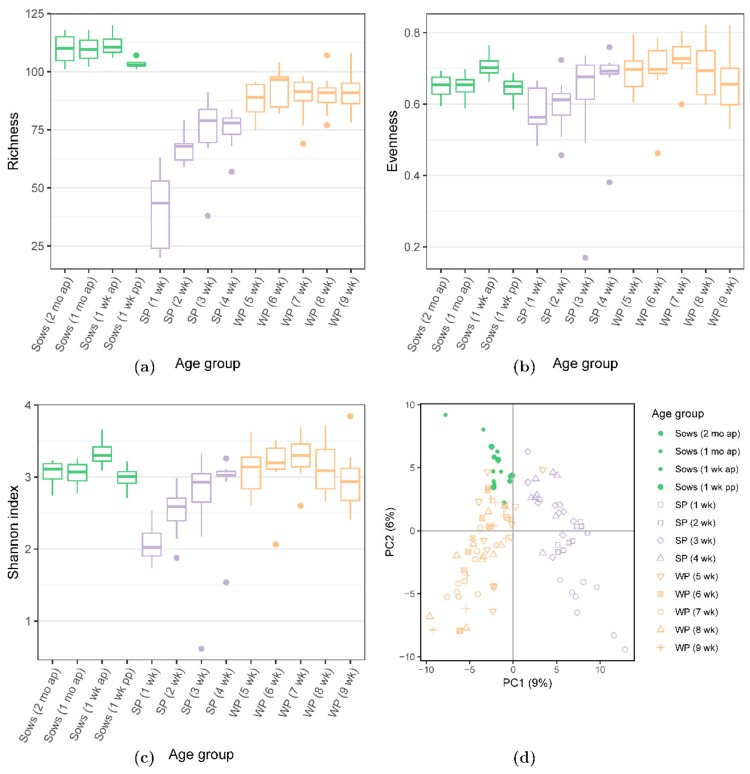
Diversity shown as (**a**) richness, (**b**) evenness, and (**c**) Shannon indices, and (**d**) a principal component analysis performed using the relative abundance of faecal microbial taxa in sows (green box plots), suckling (violet box plots) and weaned piglets (orange box plots) over time, as analysed by the 16S rDNA sequencing. Dots in figures (**a**–**c**) indicate outliers. ap: antepartum; pp: postpartum.

Microbial diversity as represented by richness, evenness, and the Shannon indices was lower in the suckling, but higher in the weaned piglets which were similar to those of the sows (Figure 2a–c). The PCA of the pig microbiome showed divergence by the age of the animals, clustering the weaned piglets closely with sows; whilst the suckling piglets were clustered more distantly from the weaned piglets and sows (Figure 2d). Comparison of the individual samples using the unweighted and weighted UniFrac distances showed the clustering by age groups (sows, suckling piglets, weaned piglets) (Supplementary Figure S1a,b).

### 3.3. C. difficile Colonisation

The presence of *C. difficile* was observed in each of the four sows during the sampling period, albeit at a low concentration (mean log_10_ 3.10 ± 0.64 copy number/g faeces). One month before farrowing, *C. difficile* was only found in one sow at a concentration of log_10_ 2.26 copy number/g of faeces. Meanwhile, one week before farrowing, only one sow carried quantifiable *C. difficile* at a concentration of log_10_ 3.00 copy number/g of faeces. Subsequently, one week after farrowing, two sows had *C. difficile* at a mean concentration of log_10_ 3.56 ± 0.14 copy number/g of faeces. Enrichment of *C. difficile* negative samples revealed the presence of this bacterium in two sows at two different sampling periods each, i.e., one month and one week before farrowing (Supplementary Table S2).

**Figure 3:**
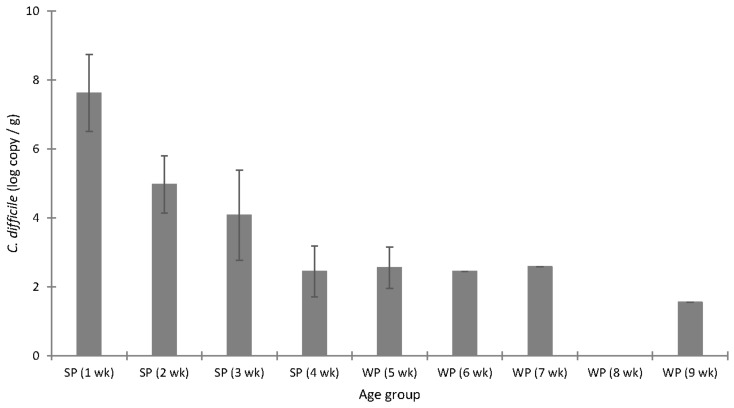
Concentration (mean ± SD, log_10_ copy/g) of *C. difficile* in faecal samples from the offspring (*n* = 8–10) sampled weekly from one until nine weeks of age and analysed using qPCR, without previous sample enrichment. SP: suckling piglets; WP: weaned piglets.

In the first two weeks of life, all piglets harboured *C. difficile* and at high concentrations up to log_10_ 9.29 copy number/g faeces. The concentration of *C. difficile* gradually decreased as the piglets aged (Figure 3).

*C. difficile* prevalence was 86% before and 97% after enrichment in the suckling piglets, and 12% before and 53% after enrichment in the weaned piglets. Overall, out of 48 offspring faecal samples that were negative for *C. difficile* from the first detection attempt using qPCR, a total of 23 turned positive (48%) after enrichment in growth media followed by qPCR detection, confirming the presence of this species in the gut (Table 1).

### 3.4. Association of the Gut Microbiota and C. difficile in Suckling Piglets

The correlation analyses revealed interactions of certain gut bacteria and *C. difficile* abundance in the suckling piglets. Notably, bacteria belonging to *Ruminococcus* 2 spp., *Christensenellaceae* R7, *Ruminococcaceae* UCG 002 and 010, *Coprococcus* 3 spp., and *Prevotellaceae* UCG 004 amongst others, were negatively associated with the level of *C. difficile*. On the other hand, bacterial taxa of *Globicatella* spp., *Pasteurella* spp., *Clostridioides* spp. (the majority of which is represented by *C. difficile* in SILVA SSU database), *Clostridium* sensu stricto 2, *Peptostreptococcus* spp., *Enterococcus* spp., *Staphylococcus* spp., and *Moraxella* spp. positively correlated with the concentration of *C. difficile* in suckling piglets (Table 2). Microbial diversity indices, such as Shannon and richness, were negatively associated with *C. difficile* counts in the suckling piglets (rho = −0.4217, *p* = 0.0181 and rho = −0.6093, *p* = 0.0003, respectively).

## 4. Discussion

The gut microbiota is characterised by dynamic development after birth with certain bacteria temporarily dominating the intestinal tract. Using molecular tools, we followed the gut microbial succession and monitored colonisation development of *C. difficile* in pigs from the first until the ninth week of life. Notably, we focused on microbiota-*C. difficile* interrelations with regard to the phenomenon termed “colonisation resistance”.

We found that in the first week of life, the gut microbiota was relatively simple and dominated by bacteria belonging to *Lactobacillus* spp., *Clostridium* sensu stricto 1 spp., *Streptococcus* spp., *Bacteroides* spp., and *Escherichia-Shigella* taxa. Although suckling piglets only shared a few bacterial taxa with sows, it was most likely that these bacterial settlers originated from the mother sow, acquired from birth canal, skin, milk, and from the surrounding environment. Similar reports on the early establishment of gut microbiota in pigs have been previously observed, where the pioneer colonisers included clostridia, enterobacteria, enterococci, streptococci, and peptostreptococci; whereas, lactobacilli and other species became predominant afterwards [1,28,29]. Introduction of solid feed during weaning reshapes the establishment of the bacterial community that began during the suckling period, and it has been associated with high rates of diarrhoea and mortality, mainly due to the overgrowth of pathogenic enterobacteria [5,29,30]. Interestingly, the gut microbiota of suckling piglets was characterised by low diversity, indicating an immature and developing micro-ecosystem. Low microbial diversity may be one of the factors that contribute to the succession of gut pathogens in early life, such as *C. difficile*. Low microbial competition may open a niche for opportunistic pathogens and predispose to disease development and/or pathogen dissemination in the environment. In the present study, we found that suckling piglets were highly colonised by *C. difficile* with regards to concentration and prevalence. The most likely source of *C. difficile* for the offspring was the mother sow where *C. difficile* spores and their toxins were detected during the gestation and lactation periods [9,10]. Here, we also report that the sow faeces contained *C. difficile*. It appeared that a low level of *C. difficile* in sows was sufficient to colonise the piglet gut successfully. However, the environmental dissemination of *C. difficile* cannot be overruled [11,12].

As the piglets aged, the concentration, as well as the prevalence of *C. difficile*-positive piglets, gradually decreased and, at the same time, the microbial diversity increased. Similarly, a decrease in the shedding load of *C. difficile* with age could be observed in calves [31]. We also found that a decrease in the *C. difficile* level was inversely associated with microbial diversity indices in suckling piglets. Specifically, *C. difficile* counts were negatively correlated with an increase in the abundance of bacteria specialised in carbohydrate degradation and short-chain-fatty-acid production in the hindgut of suckling piglets. Since all the piglets were colonised by *C. difficile* during the first two weeks of life, it was not possible to observe divergent microbiota development patterns in the animals that were colonised versus the animals that were not colonised by *C. difficile*. These results agreed with our previous observations regarding a very short time frame for colonisation by *C. difficile*, including toxigenic ribotypes [10]. Moreover, these data are very interesting and support the fact that microbial diversity is one of the factors that protects the host from *C. difficile* outgrowth and the risk for infection development. A phenomenon termed “colonisation resistance,” where other bacteria in the developing ecosystem replace *C. difficile* could contribute to the primary protection of the host from CDI. Interestingly, it has been shown in humans that *Clostridium scindens* can outcompete *C. difficile* and prevent or ameliorate CDI [32]. Moreover, non-pathogenic or less virulent *C. difficile* ribotypes, which are natural colonisers of neonatal piglets, may successfully outcompete the toxigenic ribotypes, as we have previously observed in co-culture in vitro [33], and where other authors have observed in pigs and hamsters [34,35]. In addition, increased competition for nutrients accompanying the production of short chain fatty acids by carbohydrate-fermenting commensal microbiota could have a negative impact on the succession of *C. difficile* in the hindgut, as was demonstrated in a previous in vitro study [36]. On the other hand, any disruption to the natural colonisation process, for example, by introducing formula feeding or antibiotic therapy has been linked with lower microbial diversity and CDI development in both piglets and humans [37,38]. Therefore, undisturbed colonisation with the commensal microbiota could provide an effective first line of defence against opportunistic pathogens such as *C. difficile*. However, the successful colonisation by toxigenic *C. difficile* may lead to gut intoxication and CDI development in some piglets. Interestingly, not all piglets containing a high concentration of toxins in the gut will develop CDI [10]. The reasons for spontaneous outbreaks of CDI in piglets are mostly unknown. The CDI aetiology is most likely multifactorial, and the contribution of other pathogens to the infection development or host-specific factors cannot be ignored. We have found that the level of *C. difficile* was positively associated with the bacteria of *Staphylococcus* spp., *Globicatella* spp., *Pasteurella* spp., or *Peptostreptococcus* spp. amongst others, where the species strains are known swine pathogens. Therefore, infection with *C. difficile* could contribute to the development of co-infections by opening new niches to cohabiting pathogens. On the other hand, pathogenic bacteria could indirectly favour the growth of *C. difficile* by nutrient release from damaged gut tissues during infection processes. However, these scenarios still need to be explored in more detail. Nonetheless, as a host-specific factor, sufficient antibody titres against toxins transferred from the mother sow to the offspring through milk may be crucial in the protection against gut intoxication and CDI development [33,39,40] once *C. difficile* colonisation is established in the gut.

Notably, almost half of the weaned-piglet-faeces that were negative for *C. difficile* (detected by qPCR) turned out to be positive after sample enrichment. The differences in *C. difficile* prevalence before and after sample enrichment indicated that this bacterium, although often reported as being absent in biological specimens, may still inhabit the host and shed spores into the environment. This may be a serious threat to the animals, farm workers, and the environment, in terms of antibiotic multi-resistance and dissemination of hyper-virulent ribotypes. Such findings are significant for the veterinary and clinical diagnostics of *C. difficile*, and they indicate that the prevalence of this bacterium may be underestimated when detection is performed without the sample enrichment medium that was used in this study. Our enrichment medium consisted of brain-heart-infusion, yeast extract, l-cysteine, sodium taurocholate for the induction of spore germination, and the antibiotics cycloserine and cefoxitin, that are known to inhibit a vast number of gram-positive and gram-negative bacteria without having an impact on *C. difficile* [41]. This approach allowed us to propagate *C. difficile* to a sufficient number of vegetative cells that became detectable using the qPCR method, thereby turning seemingly negative samples into positive ones. Indeed, the qPCR method has been one of the principal molecular techniques used to detect and quantify the bacteria of interest in faecal samples. The majority of studies describing *C. difficile* detection by qPCR in clinical specimens have not applied the sample enrichment method. Moreover, clostridia form spores that are considered the most challenging regarding nucleic acid extraction and molecular quantification of spore-forming bacteria. We have already successfully improved the DNA extraction method from spore-forming bacteria, such as *C. difficile*, and were able to decrease the detection limit by a log unit, compared to standard DNA extraction methods [17]. This technique improvement was applied in the present study to extract the microbial DNA for sequencing and qPCR, and it significantly improved the signals from bacterial genomes. Indeed, nucleic acid extraction without special treatment for bacterial spores may underestimate the *C. difficile* cell numbers.

## 5. Conclusions

This study shows that the gut microbiota seems to set conditions for colonisation resistance against *C. difficile* in the offspring. However, additional host-factors cannot be overruled. The phenomenon of colonisation resistance and susceptibility to *C. difficile* infection in suckling piglets requires more focus. In the future, the modulation of gut microbiota could be an attractive approach to controlling *C. difficile* colonisation in piglets.

## Figures and Tables

**Table 1:** Prevalence (%) of *C. difficile* in the faecal samples from the offspring (suckling and weaned piglets) (*n* = 8–10) sampled weekly from one until nine weeks of age. Faecal samples negative for *C. difficile* from qPCR were enriched in a specific growth media, followed by DNA extraction and qPCR.

Age (Week)	% of Positive Samples	
	Before Enrichment	After Enrichment
1	100	100
2	100	100
3	90	90
4	56	100
5	30	80
6	10	60
7	10	60
8	0	10
9	11	56

**Table 2:** Significant associations between the abundance of specific bacterial taxa (16S rDNA sequencing) and *C. difficile* counts (qPCR) in the faecal samples from suckling piglets that were sampled weekly from one until nine weeks of age.

Taxon	Pearson’s *r* (*n* = 31)	*p*
*Ruminococcus* 2 spp.	−0.5960	0.0004
*Christensenellaceae* R7	−0.5936	0.0004
*Romboutsia* spp.	−0.5936	0.0004
*Marvinbryantia* spp.	−0.5669	0.0009
*Ruminococcaceae* UCG 002	−0.5566	0.0012
*Fournierella* spp.	−0.5488	0.0014
*Ruminococcaceae* UCG 010	−0.5296	0.0022
*Terrisporobacter* spp.	−0.5286	0.0023
*Coprococcus* 3 spp.	−0.5213	0.0026
*Hydrogenoanaerobacterium* spp.	−0.5193	0.0028
*Prevotellaceae* UCG 004	−0.5188	0.0028
*Corynebacterium* 1 spp.	−0.5163	0.0029
*Globicatella* spp.	0.5025	0.0040
*Pasteurella* spp.	0.5257	0.0024
*Clostridioides* spp.	0.5381	0.0018
*Clostridium* sensu stricto 2 spp.	0.5838	0.0006
*Peptostreptococcus* spp.	0.6064	0.0003
*Enterococcus* spp.	0.6272	0.0002
*Staphylococcus* spp.	0.6428	9.6402 × 10^−5^
*Moraxella* spp.	0.6447	9.0628 × 10^−5^

## Supplementary Materials

The following are available online at https://www.mdpi.com/2076-2607/7/8/218/s1. Table S1. List of bacterial taxa that are unique and shared between sows, suckling and weaned piglets, as analysed by 16S rDNA sequencing. Table S2. Concentration of *C. difficile* in faecal samples from the sows sampled during the periparturient period and analysed using qPCR without and with previous sample enrichment (faecal samples negative for *C. difficile* from qPCR were enriched in a specific growth media, followed by DNA extraction and qPCR). Figure S1. UniFrac (**a**) unweighted and (**b**) weighted phylogenetic distance analyses of the faecal microbial taxa in sows, suckling and weaned piglets over time, as analysed by 16S rDNA sequencing. wk: week; mo: month; ap: antepartum; pp: postpartum.
