# Supplementary material for: Developing Gut Microbiota Exerts Colonisation Resistance to Clostridium (syn. Clostridioides) difficile in Piglets
Source: Microorganisms. 2019 Jul 26;7(8):218. doi: 10.3390/microorganisms7080218 (PMC6723027; doi:10.3390/microorganisms7080218)
Supplement: Supplementary file 1 [file microorganisms-07-00218-s001.zip › Supplementary Figure 1b.pdf]

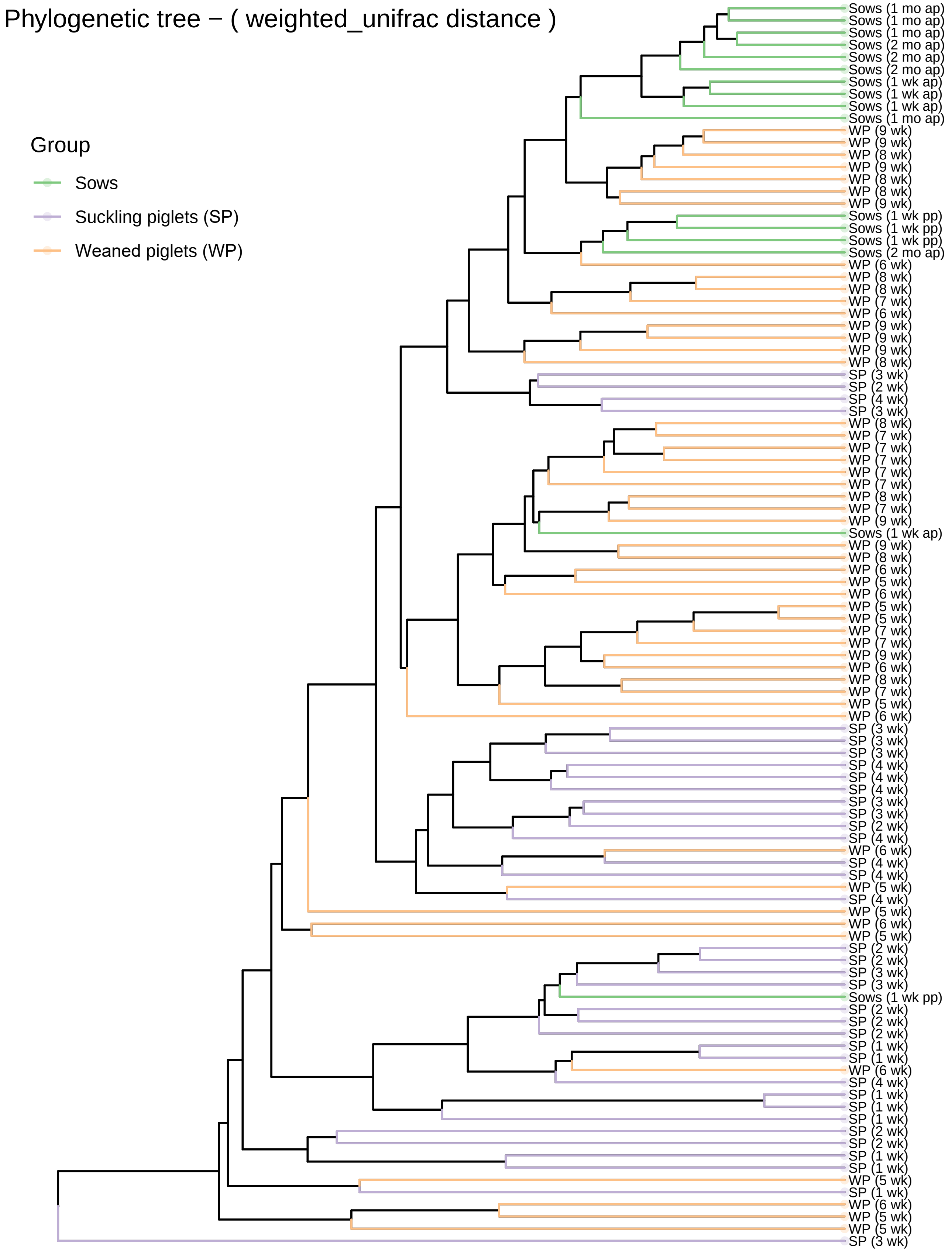
Phylogenetic tree − (weighted_unifrac distance)
Group
Sows
Suckling piglets (SP)
Weaned piglets (WP)
Sows (1 mo ap)
Sows (1 mo ap)
Sows (1 mo ap)
Sows (2 mo ap)
Sows (2 mo ap)
Sows (2 mo ap)
Sows (1 wk ap)
Sows (1 wk ap)
Sows (1 wk ap)
Sows (1 mo ap)
WP (9 wk)
WP (9 wk)
WP (8 wk)
WP (9 wk)
WP (8 wk)
WP (8 wk)
WP (9 wk)
Sows (1 wk pp)
Sows (1 wk pp)
Sows (1 wk pp)
Sows (2 mo ap)
WP (6 wk)
WP (8 wk)
WP (8 wk)
WP (7 wk)
WP (6 wk)
WP (9 wk)
WP (9 wk)
WP (9 wk)
WP (8 wk)
SP (3 wk)
SP (2 wk)
SP (4 wk)
SP (3 wk)
WP (8 wk)
WP (7 wk)
WP (7 wk)
WP (7 wk)
WP (7 wk)
WP (7 wk)
WP (8 wk)
WP (7 wk)
WP (9 wk)
Sows (1 wk ap)
WP (9 wk)
WP (8 wk)
WP (6 wk)
WP (5 wk)
WP (6 wk)
WP (5 wk)
WP (5 wk)
WP (7 wk)
WP (7 wk)
WP (9 wk)
WP (6 wk)
WP (8 wk)
WP (7 wk)
WP (5 wk)
WP (6 wk)
SP (3 wk)
SP (3 wk)
SP (3 wk)
SP (4 wk)
SP (4 wk)
SP (4 wk)
SP (3 wk)
SP (3 wk)
SP (2 wk)
SP (4 wk)
WP (6 wk)
SP (4 wk)
SP (4 wk)
WP (5 wk)
SP (4 wk)
WP (5 wk)
WP (6 wk)
WP (5 wk)
SP (2 wk)
SP (2 wk)
SP (3 wk)
SP (3 wk)
Sows (1 wk pp)
SP (2 wk)
SP (2 wk)
SP (2 wk)
SP (1 wk)
SP (1 wk)
WP (6 wk)
SP (4 wk)
SP (1 wk)
SP (1 wk)
SP (1 wk)
SP (2 wk)
SP (2 wk)
SP (1 wk)
SP (1 wk)
WP (5 wk)
SP (1 wk)
WP (6 wk)
WP (5 wk)
WP (5 wk)
SP (3 wk)
