# Supplementary figures and images for: Developing Gut Microbiota Exerts Colonisation Resistance to Clostridium (syn. Clostridioides) difficile in Piglets
Source: Microorganisms. 2019 Jul 26;7(8):218. doi: 10.3390/microorganisms7080218 (PMC6723027; doi:10.3390/microorganisms7080218)

## Phylogenetic tree – ( unweighted\_unifrac distance )

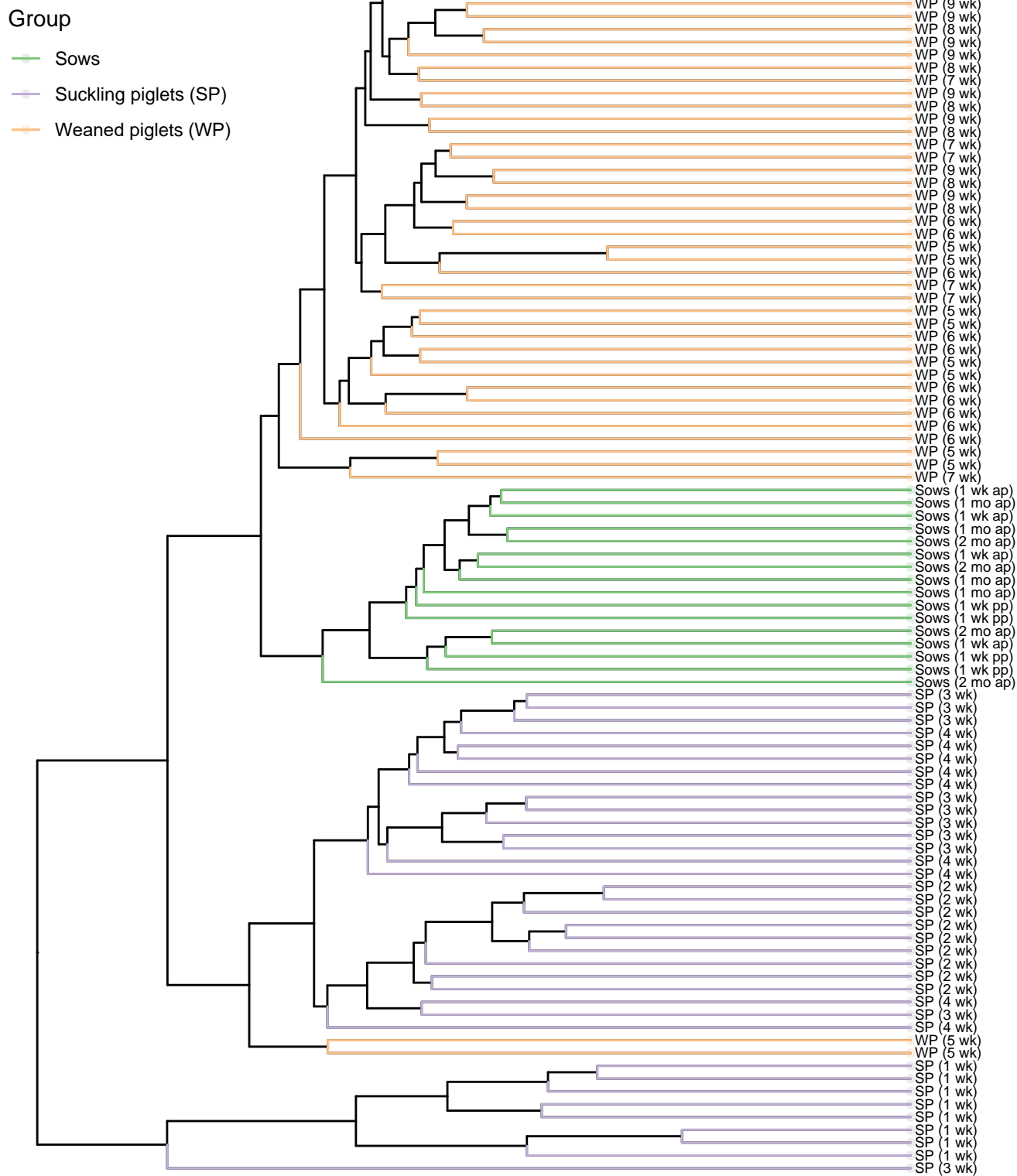

Supplement: Supplementary file 1 [file microorganisms-07-00218-s001.zip › Supplementary Figure 1a.pdf]
